# Microfluidics-integrated spaceflight hardware for measuring muscle strength of *Caenorhabditis elegans* on the International Space Station

**DOI:** 10.1038/s41526-022-00241-4

**Published:** 2022-11-07

**Authors:** Purushottam Soni, Taslim Anupom, Leila Lesanpezeshki, Mizanur Rahman, Jennifer E. Hewitt, Matthew Vellone, Louis Stodieck, Jerzy Blawzdziewicz, Nathaniel J. Szewczyk, Siva A. Vanapalli

**Affiliations:** 1 Department of Chemical Engineering, Texas Tech University, Lubbock, TX 79409 USA; 2 Department of Electrical Engineering, Texas Tech University, Lubbock, TX 79409 USA; 3 BioServe Space Technologies, Boulder, CO 80303 USA; 4 Department of Mechanical Engineering, Texas Tech University, Lubbock, TX 79409 USA; 5 Ohio Musculoskeletal and Neurological Institute and Department of Biomedical Sciences, Ohio University, Athens, OH 43147 USA

**Keywords:** Human behaviour, Biological techniques

## Abstract

*Caenorhabditis elegans* is a low-cost genetic model that has been flown to the International Space Station to investigate the influence of microgravity on changes in the expression of genes involved in muscle maintenance. These studies showed that genes that encode muscle attachment complexes have decreased expression under microgravity. However, it remains to be answered whether the decreased expression leads to concomitant changes in animal muscle strength, specifically across multiple generations. We recently reported the NemaFlex microfluidic device for the measurement of muscle strength of *C. elegans* (Rahman et al., Lab Chip, 2018). In this study, we redesign our original NemaFlex device and integrate it with flow control hardware for spaceflight investigations considering mixed animal culture, constraints on astronaut time, crew safety, and on-orbit operations. The technical advances we have made include (i) a microfluidic device design that allows animals of a given size to be sorted from unsynchronized cultures and housed in individual chambers, (ii) a fluid handling protocol for injecting the suspension of animals into the microfluidic device that prevents channel clogging, introduction of bubbles, and crowding of animals in the chambers, and (iii) a custom-built worm-loading apparatus interfaced with the microfluidic device that allows easy manipulation of the worm suspension and prevents fluid leakage into the surrounding environment. Collectively, these technical advances enabled the development of new microfluidics-integrated hardware for spaceflight studies in *C. elegans*. Finally, we report Earth-based validation studies to test this new hardware, which has led to it being flown to the International Space Station.

## Introduction

Among the many pathophysiological changes that occur during human spaceflight, muscle atrophy is significant and remains a major impediment to deep space exploration^1,2^. The postural muscles are most affected in the microgravity environment, therefore, impacting the activities of astronauts in long-duration spaceflight missions. Even a weeklong stay at the International Space Station (ISS) can lead to degradation in muscle mass and peak power; this loss could be fatal in off-nominal landing scenarios where assistance might be unavailable. Loss of muscle strength can pose an even more serious problem for interplanetary travel, such as with a venture to Mars, which could take 200 to 300 days.

Generally, reduced workload in a microgravity environment is considered to be a primary cause of muscle loss in response to spaceflight^3–5^. However, altered metabolism also plays a significant role^6,7^. For example, atrophy in flight is not limited to antigravity muscles but also occurs in vascular smooth muscles and cardiac muscles^8–10^. To combat muscular atrophy, astronauts exercise (aerobic and resistance) for up to 2.5 h each day. Even with these current countermeasures, the loss in muscle mass is up to 20% during a 5-day to 2-week-long flight, whereas for a long-duration space mission (3 to 6 months), a loss of 30% is noted^3,11^. In most cases, muscle mass and strength can be regained within a few months to years after returning to Earth by a combination of adapted exercise and rehabilitation^11,12^. Nevertheless, muscle maintenance in space is still a major concern, and no effective preventive measures exist beyond the limited benefit obtained through exercise. Thus, it is important to determine the mechanisms responsible for the loss of muscle strength in spaceflight and develop appropriate countermeasures.

In previous studies, it was shown that alteration in gene expression (e.g., in genes encoding myosin heavy chain kinase and other cytoskeletal proteins) in response to microgravity is similar in cultured embryonic muscle cells^1^, animals (both vertebrate and invertebrate), and humans^10,13^. These similarities provide strong motivation for investigating the effect of spaceflight conditions on muscles using invertebrate animal models, thus avoiding the cost and time expense associated with experiments using vertebrates. Indeed, the invertebrate model organism, *Caenorhabditis elegans* (*C. elegans*), has been recognized as an excellent model system for space biology research and has been flown in several spaceflight investigations^14–19^.

The *C. elegans* model presents several advantages for spaceflight studies. Up to 40% of its genes are homologous to human genes^20,21^, and a large library of genetic mutants^22^ and strains with fluorescent reporters are available. The body wall muscles of *C. elegans* are functionally analogous to vertebrate muscle, as they include dense bodies and M-lines, the former being functionally equivalent to vertebrate Z-lines. In addition, the nematode is small (≈1 mm), weight is miniscule (≈1 μg), has a short lifespan (≈20 days), fast generation time (∼1 week), and is easy to grow. For spaceflight missions where payload size and weight are critical considerations, *C. elegans* experimental payloads offer a small form factor and a significant reduction in weight compared with vertebrates. Moreover, the fast reproductive cycle enables multi-generational studies to be conducted, providing an opportunity to explore physiological adaptations due to long-term habitation in space. These advantages and existing strain resources make *C. elegans* a leading invertebrate animal model for spaceflight studies.

On Earth, adult *C. elegans* cultured in liquid media tend to sediment, indicating that animals experience gravitational stresses^23,24^. In microgravity, gravitational effects are significantly weaker, which alters *C. elegans* physiology^13^. In prior spaceflight experiments with *C. elegans*, it was found that approximately 150 muscle genes were downregulated in *C. elegans* during 10 days of culture in microgravity^13,25^. Among the detected downregulated muscle genes were key motor protein genes such as MyoD and myosin heavy chain, and two genes (*unc-97* and *unc-112*) that encode members of a muscle attachment complex^13^. Members of the attachment complex (called the costamere in humans) have been shown in humans to increase expression and activity with increased mechanical load and decrease expression and activity in response to immobilization. Thus, muscle attachment genes play an important role in processes leading to muscle loss. In *C. elegans* on Earth, decreased expression of these genes via acute RNAi treatments results in movement defects and various subcellular defects within the muscle (e.g., disorganization and collapse of arrayed sarcomeres)^26^.

A key open question is if and how the changes in muscle gene expression observed in spaceflight result in decreased muscle strength. While decreased force production is documented for astronauts, it is yet to be demonstrated for *C. elegans* cultured in microgravity. Several studies have used elastomeric micropillars to measure muscle forces in *C. elegans*^27–29^, and we have recently standardized a microfluidic device and approach for obtaining the maximum exertable force, providing a measure of nematode muscle strength. This strength measurement apparatus is called NemaFlex^30,31^ and consists of a microfluidic chamber with microfabricated deformable pillars. The force produced by a nematode moving between the pillars is evaluated from the pillar deflections observed under a microscope. The NemaFlex device and the force measurement protocol have been optimized such that muscle strength is independent of behavior and gait. The device is also capable of detecting strength differences in mutants with defects in muscle genes^31,32^. The NemaFlex device is miniaturized and simple to use, and thus has potential for *C. elegans* spaceflight experiments.

In this study, we address technical challenges for conducting multi-generational *C. elegans* strength measurements in microgravity. These technical challenges include culturing of animals across several generations, obtaining nearly age-synchronized animals of a given size for strength measurements, establishment of an imaging protocol for recording pillar deflections, and development of an on-orbit protocol for experimentation that minimizes crew time and ensures astronaut safety on the ISS. Addressing these technical challenges, we report a reconfigured spaceflight ready NemaFlex device (referred to as NemaFlex-S), new protocols for experimentation with *C. elegans*, and a custom-designed hardware for on-orbit operation of NemaFlex-S on the ISS. This integrated technology and protocols enabled its successful operation by astronauts on the ISS producing significant visual data of *C. elegans* across multiple generations.

## Results

### *C. elegans* culture for multigenerational spaceflight studies

The goal of our spaceflight experiment on ISS is to measure the strength of adult wild-type *C. elegans* at 4-time points across an 8-week period corresponding to 8 generations. This study design allows the evaluation of potential changes in muscle strength due to short and long-term muscle adaptation to the microgravity environment. In contrast to conducting experiments on Earth, spaceflight experiments present unique constraints that deserve special consideration. These constraints include crew time for conducting the experiments, maintaining sterile culture conditions, and the safety of the crew members and workstation at ISS. Therefore, while developing culturing and fluid handling protocols, the microfluidic device, and the worm loading apparatus, we considered these spaceflight constraints.

The first challenge was to culture *C. elegans* for several generations on ISS. Table 1 lists the different space-flight missions with *C. elegans*, the culturing hardware, and the food source used in these studies. In typical laboratory conditions on Earth, *C. elegans* is cultured on nematode growth media (NGM) plates. Culturing *C. elegans* on NGM plates is generally labor-intensive as it requires manual picking of animals and transfers to new plates to maintain synchronized animal populations. Such laborious tasks consume significant crew time; therefore, the culture of *C. elegans* on NGM plates remains impractical for multigenerational studies in space.

**Table 1:** Summary of previous space missions with *C. elegans*.

Mission	Experiment duration	Culture hardware	Food source/diet	Image-based quantitative phenotyping on ISS
STS-42, 1992^36,37^	8 days	Agar Plate	*E. Coli OP50*	No
STS-76, 1996^60^	9 days	M9 buffer	No Food	No
STS-95, 1998	10 days	---	----	No
STS-107, 2003^38^	16 days	Agar and CeMM Plates	UV killed *E. coli OP50* and CeMM both	No
ICE-FIRST, 2004^52^	10 days	Polyethylene bags	CeMM	No
CSI-1, 2006^58^	6 months	Opticells	CeMM	Yes, for development and behaviors
CERISE, 2009^17,53^	8 days	Polyethylene bag	*E. Coli OP50*	Yes, videos to quantify worm behaviors
Shenzhou-8 Mission, 2011^39^	17 days	Agar Plate	*E Coli* OP50	No
Space Aging, 2015	70 days	culture chambers	CeMM	Yes, No published result
Epigenetics, 2015^61^	17 days	NIPRO bag	*E. Coli OP50*	No
Nematode Muscle, 2015^62^	4 days	NIPRO bag	*E. Coli OP50*	No
Molecular Muscle experiment, 2018^63^	7 days	Polyethylene bag	Freeze-dried *OP50*	No
This Study, 2021	49 days	Fluorinated Ethylene Propylene bag	CeMM	Yes, to quantify muscle strength and behaviors

An additional constraint is the maintenance of bacterial food source for culturing worms. Although non-pathogenic *E. coli* OP50 is typically used as the food source, maintenance of defined bacterial population densities across the 8-week duration of our spaceflight experiment may present challenges since high bacterial density might limit oxygen availability for nematodes. *C. elegans* on NGM plates are used mostly in simulated microgravity on Earth^33–35^. *C. elegans* were flown to space on a standard agar-based nematode growth medium for a short duration of 10 to 20 days with different space missions^36–39^ as listed in Table 1. But the missions do not involve imaging onboard, therefore all the behavioral studies were conducted postflight.

To overcome the issues discussed above, the wild-type worms were cultured in chemically defined liquid *C. elegans* maintenance media (CeMM)^40^. The safety and low-maintenance requirements make CeMM robust and appropriate media for multigenerational cultures on board the International Space Station. However, previous experiments show there are some differences between nematodes grown using NGM and CeMM^40,41^. In particular, *C. elegans* development is slower, progeny production is slower, and lifespan is increased in *C. elegans* cultured in CeMM compared with those cultured using NGM^41^. In addition, the culture vessels used to maintain *C. elegans* could impact their growth. Therefore, there was a need to optimize culture protocols in CeMM and in spaceflight compatible culture vessels to achieve consistent culture densities across multiple generations.

**Fig. 1:**
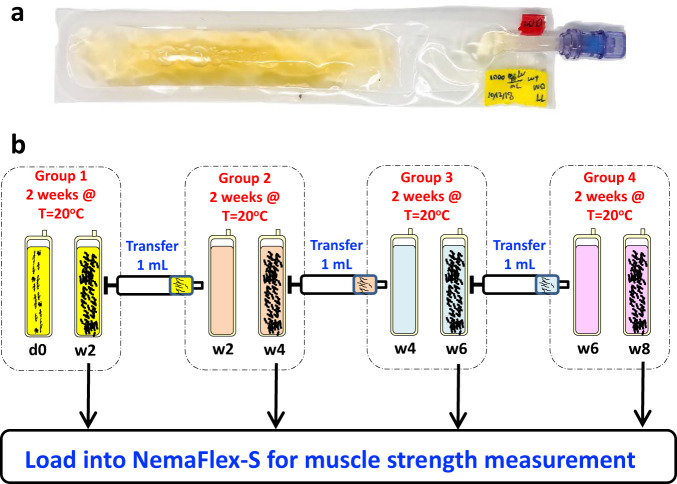
*C. elegans* culture in CeMM and the multigenerational culture protocol. **a** An FEP bag containing wildtype *C. elegans* culture in CeMM. Image used in this figure was captured at TTU. **b** A schematic protocol for culturing *C. elegans* for multigenerational studies over an 8-week period. Here, d0 represents day 0 (start of the culture), and w2, w4, w6, and w8 represent week 2, week 4, week 6, and week 8, respectively.

The worms were maintained in CeMM in biocompatible Fluorinated Ethylene Propylene (FEP) bags (Fig. 1a). These bags provide a solution for culturing *C. elegans* in liquid media since they are highly permeable to oxygen and carbon dioxide while remaining impermeable to water^42^. An efficient and robust protocol was developed for culturing the animals to maintain the desired density across several generations to prevent overcrowding and ensure nutrient availability. A schematic of the culturing protocol for multigenerational studies *in C. elegans* is depicted in Fig. 1b. Primary bags (group 1) with approximately 1000 larvae/mL were prepared at day 0 and incubated at 20 ± 1 °C for 2 weeks. On week 2, a specified aliquot (1 mL) of the media with worms was transferred from this bag to a new bag that was prefilled with 19 mL of fresh CeMM. The new bag was incubated at 20 ± 1 °C, and the old bag was used for loading the worms into the NemaFlex-S device. The same culturing protocol was followed every other week for the remaining generations. No transfer was made on week 8.

**Fig. 2:**
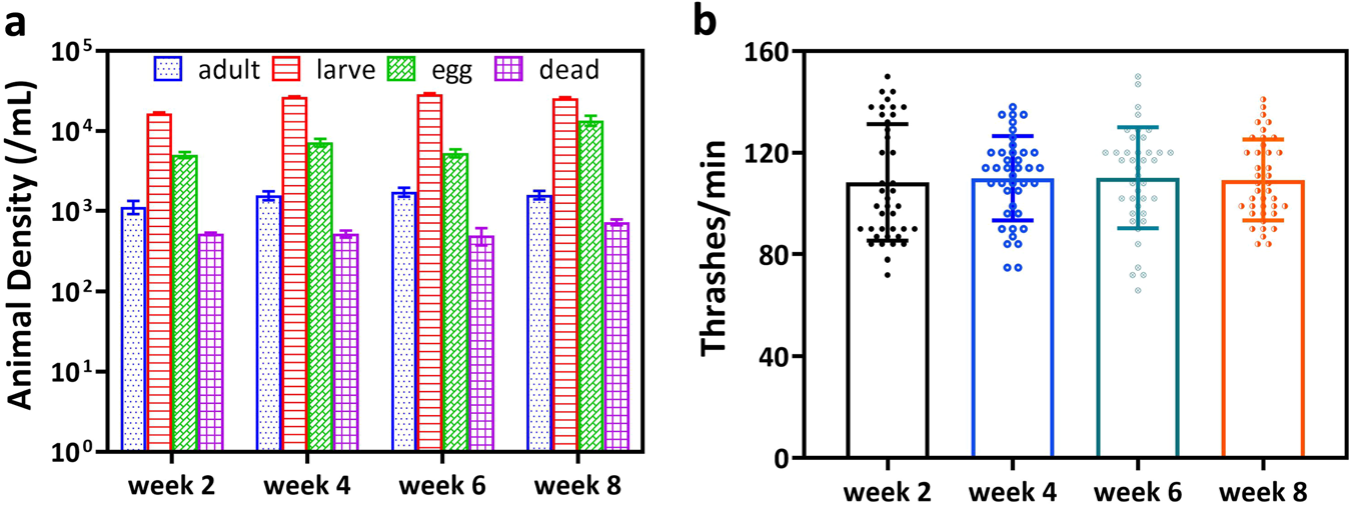
Characterization of culture growth and health across the eight-week multi-generational experiment. **a** Characterization of the culture density including adults, larvae, eggs, and dead animals. **b** Swim-induced thrashing frequency of gravid adults as a measure of their locomotory health (Supplementary Table 1). Error bars represent standard deviation. All the data pass the normality test. There is no significant difference between thrashing frequency as calculated by one-way ANOVA, *P* ≥ 0.7.

The efficacy of the multigenerational culturing protocol discussed in Fig. 1b was evaluated by measuring animal density and locomotory health during the 8-week multigeneration experiment. Since the culture was not age-synchronized, we measured the density of adults, larvae, eggs, and dead animals in the FEP bags. In parallel, we also measured the thrashing frequency of gravid adults as a health measure. Both these measurements were conducted on weeks 2, 4, 6 and 8. As illustrated in Fig. 2a, the culture consisted of more than 1000 adults/mL, the approximate density needed for optimized loading into the NemaFlex-S device. The culture was also found to have several thousands of larvae and eggs, which shows good reproductive health and overall health of the animals in the culture. With regards to locomotory health, we find that the thrashing frequency is the same across all four-time points (Fig. 2b). Thus, the overall culture health is not compromised over the 8-week multigenerational experiment.

### Design of the NemaFlex-S microfluidic device

Our concept for designing a spaceflight ready microfluidic device involved ideally housing individual animals in small chambers so that the astronaut performing the imaging can easily locate and record videos of animals, thereby reducing crew time. In recent years efforts have been made in studying the behavior of *C. elegans* in longitudinal chips^43–46^, worms were loaded automatically into these chips. Prior literature provides design guidance on how to house individual *C. elegans* in microfluidic chambers, however, these devices do not meet the needs of multigenerational space-flight studies. For example, Chung et al.^43^ and Le et al.^44^ designed a microfluidic device that had an array of connected chambers with tapered necks for individual animals to enter and be housed. Although these devices can successfully house individual animals, they were optimized for age-synchronized animals and not for mixed populations where progeny needs to be removed. Moreover, the chambers were not configured with flexible pillars, which makes them unsuitable for strength measurements. Additionally, in these Earth-based studies, worms were cultured on plates and then transferred to liquid media. Transferring the liquid culture in a microfluidic device in an open manner is not safe as it can lead to contamination of the workstation at ISS and possibly cause health hazards due to the presence of bacterial food.

Building on this prior work, we considered additional criteria relevant to our spaceflight experiment that led to technical advances in engineering the NemaFlex-S device. These additional criteria include: (i) given that the culturing protocol generates a mixed population of animals, the device design should be capable of sorting and obtaining nearly age- and size-matched animals since muscle strength of *C. elegans* can vary with developmental stage and body size^30^, (ii) the pillar diameter and spacing should be modified to accommodate CeMM-grown animals, which are skinnier than bacteria-fed worms for whom the original NemaFlex deflectable pillar geometry was optimized, (iii) maximize the number of chambers with 1 to 2 animals so that the image-processing code can detect pillar deformations and cleanly associate it with the corresponding animal making those deflections, (iv) minimize entry of air bubbles or plugs into the pillar arena that can negatively impact animal loading and image processing, which ultimately can reduce collection of useful animal data from the space experiment, (v) allow a means for the crew on orbit to easily focus the microscope objective on the pillar rim which is crucial for the software to auto-detect the deflections, and (vi) establish a relatively simple chip-to-world interface so that the crew time is reduced for loading the animals into the individual pillar chambers.

**Fig. 3:**
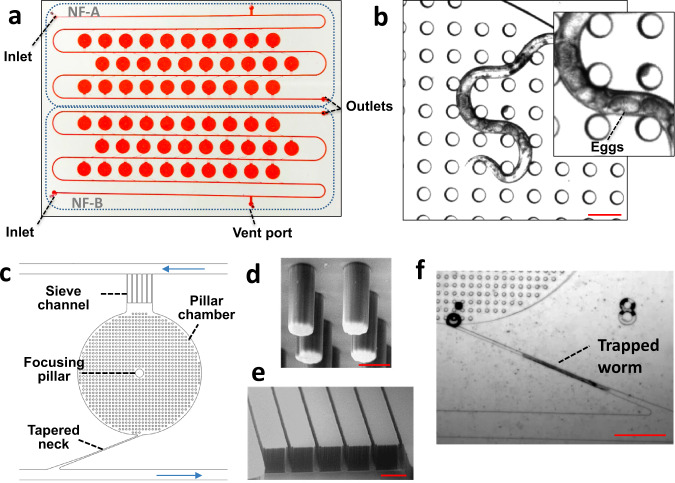
Design of the NemaFlex-S device for strength measurement of *C. elegans*. **a** An actual image of the NemaFlex-S device with two identical sections NF-A and NF-B, the device is filled with red food dye for better visualization of the salient features. **b** Image of a gravid adult crawling in the pilar chamber. The zoomed inset image shows the eggs inside the worm. Scale bar, 100 µm. **c** Design of the individual pillar chamber. The chamber is connected to the flow channel (highlighted with blue arrows) with a tapered neck for trapping the worms and with sieve channels for removing the progenies. **d** Scanning electron microscopy (SEM) image of deformable micropillars. Scale bar, 50 µm. **e** SEM image of sieve channels. Scale bar, 100 µm. **f** Image of an adult worm trapped in the neck Scale bar, 500 µm. See Supplementary Video for trapping and loading of an adult into the chamber. Images used in this figure were captured at TTU.

The layout of the NemaFlex-S device is shown in Fig. 3a and consists of two identical sections of 30 chambers, each with pillars. The presence of 2 separate sections allows redundancy in case one section clogs and also reduces pressure drop during animal loading. Each section has a single inlet, a single outlet, and a side vent port to remove air bubbles. The pillar chamber has a diameter of 3 mm to allow the 1 mm long adults to move freely within the pillars arranged as a square lattice (Fig. 3b). The design of the pillar lattice was based on the body diameter of CeMM-grown adults and the optimal body confinement between pillars needed to induce maximum exertable force^30^. The body diameter of the adult worms grown in CeMM is *D* ≈45 μm, which is about half the diameter of animals grown on NGM plates with *E. coli* OP50 diet. Therefore, pillars of diameter 40 μm were designed along with the pillar gap being ≈40 μm so that the body confinement of ≈1 is within the admissible range^30^. The pillar chamber height was designed to be ≈100 μm and the pillar height to be ≈80 μm, providing a clearance of ≈20 μm between the pillar tip and the chamber floor. To facilitate easy selection of the imaging focal plane by the crew, a focusing pillar of 200 µm in diameter was introduced at the center of the chamber (Fig. 3c).

To obtain animals of a specific age from the mixed population in FEP bags, we focused on sorting gravid adults since it allows the crew to visually confirm these egg-bearing animals (Fig. 3b). An on-chip strategy for size-based sorting of gravid adults was implemented by adding two key geometric features to the pillar chambers – tapered necks and sieve channels, as shown in Fig. 3c–e. The principle underlying these geometric features is that an animal of a given size is arrested in the tapered neck, while animals below this size are washed out from the sieve channel. The neck was designed to be ≈2.2 mm long with the minimum width being ≈25 μm such that the 1 mm long adult remains trapped in the neck until the flow rate is increased to push the trapped animal inside the chamber. The sieve channels separated by ≈20 μm at the end of the chamber serve the dual purpose of retaining the adult in the chamber but allowing a path for the washout of larvae and eggs^43,47^. The high-resolution images of the pillars and sieve channels are shown in Fig. 3d and Fig. 3e, respectively. Since the chambers are connected to the flow channel via the tapered neck and sieve channels, they provide a low resistance path for the worm suspension to flow. Gravid adults passing through the flow channel tend to follow the low resistance path and become trapped in the necks (Fig. 3f). Once most of the necks are occupied, the adults can be pushed into the chambers by setting the pump to higher flow rates (See Supplementary Movie 1), as discussed later in the paper.

### Design of the worm-loading apparatus

The worm suspension in FEP bags and the NemaFlex-S device need to be integrated in a facile manner to allow the crew to load worms in the pillared chambers. This bag-to-chip integration was achieved using a worm loading apparatus (WLA) to prevent open handling of culture that presents a biohazard on ISS. The design of the WLA addressed several requirements for the crew to conduct the experiment in an efficient and safe manner. These requirements include (i) containment of the glass-bonded microfluidic device in case of potential glass breakage, (ii) storage and dispensing of samples including waste in closed containers to avoid fluids that might float in microgravity and contaminate the ISS environment, and (iii) a flow distribution system that allowed switching of different fluids that need to be injected into the NemaFlex-S device during the loading protocol. Although such requirements are not critical for conducting microfluidic experiments on Earth, they become crucial due to crew safety and time being a priority on ISS.

**Fig. 4:**
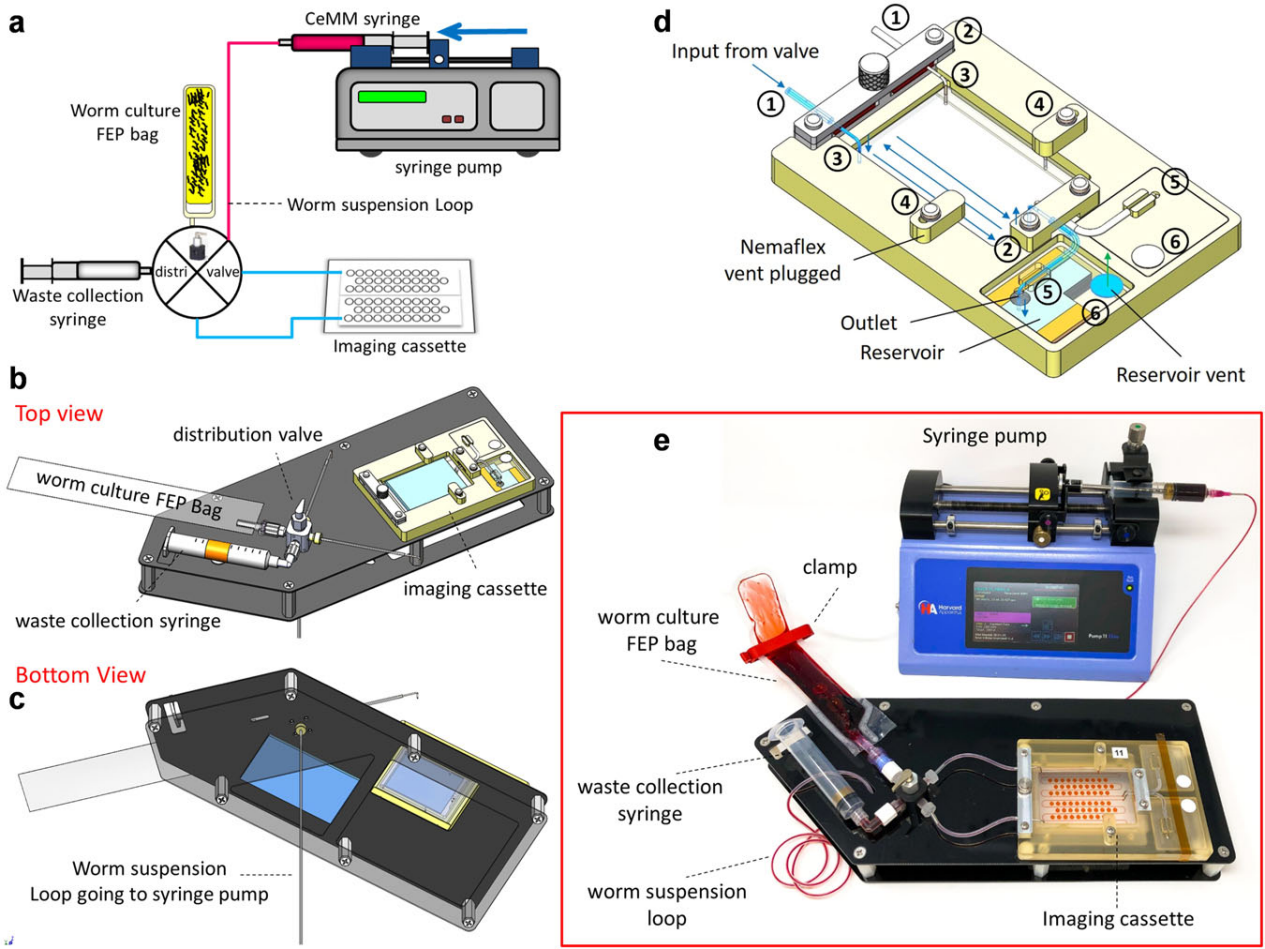
Microfluidics-integrated worm loading apparatus. **a** Schematic diagram of the worm loading apparatus (**b**) and (**c**) shows the schematic top and bottom view of the WLA board and its key component, respectively. **d** A leak proof imaging cassette for loading the worms into NemaFlex-S Cassette. The highlighted parts are: (1) inlet lines connected to the distribution valve (2) clips holding the inlet and outlet cannulas (3) L-shaped cannulas connected to the inlet of NemaFlex-S chip (4) cannula connected to air-vent purge port (5) outlet cannula opening into the waste collection chamber (6) porous membrane for releasing the air. **e** Image of the actual worm loading apparatus showing the three major hardware components – Imaging cassette, distribution valve, and the syringe pump. The worm culture bag and 10 mL waste collection syringe are connected to the distribution valve; the worm suspension loop carries either the CeMM from the media syringe or worm solution from the culture bag. The distribution valve has a feeding port (shown in fig. **c**) underneath the mounting board. Image used in this figure was captured at TTU.

The schematic of the WLA shown in Fig. 4a was designed for leak-proof and efficient loading of the worms into the NemaFlex-S device. WLA has four key hardware components: (i) an imaging cassette, (ii) a flow distribution valve, (iii) a programmable syringe pump, and (iv) a specially designed board to mount hardware components and fluid lines (Fig. 4b, c). The worm suspension loop shown in Fig. 4a goes underneath the distribution valve (see Fig. 4b, c). The imaging cassette schematic shown in Fig. 4d housed the glass-bonded microfluidic device. It was machined from polysulfone–a high-performance plastic. The retention clips serve to hold the Nemaflex-S device and the inlet/outlet cannulas. The fasteners on either side are aligned with the side vent ports to purge air and then plug the vent holes. Two waste reservoirs collect the outgoing liquid (up to 3 mL each side) from the NemaFlex-S device during the worm loading process and are vented by porous membranes. The lids to the waste chambers are designed to be adhesively attached. The inlet cannulas are connected to the distribution valve with the help of tubing, and outlet cannulas are connected to the waste reservoirs. A polydimethylsiloxane (PDMS) replica of the NemaFlex-S device of 4.25 ± 0.25 mm thickness can be integrated into the cassette. The imaging cassette was mounted on the board using Velcro^®^. The cassette can be removed after loading the worms into the NemaFlex-S device and mounted on the microscopic stage for the crew to perform imaging. The actual image of the WLA is shown in Fig. 4e.

Another crucial component of the WLA apparatus is the 4-way distribution valve. The feed to the distribution valve comes from the CeMM syringe mounted on the syringe pump (see Fig. 4a, e) via an inlet port present underneath the mounting board (see Fig. 4b, c). The length of tubing from the media syringe is predetermined to create a fluid loop that can hold a defined volume of worm suspension aspirated from the FEP culture bag. Two ports of the valve are connected to the two inlets of the imaging cassette. The remaining two ports are connected to the worm culture bag and the waste collection syringe, respectively. The direction of fluid flow can be manually controlled by turning the knob on the distribution valve. A script was programmed into the syringe pump, allowing the astronauts to click on the pump program and follow the instructions to load the animals into the NemaFlex-S device.

### Optimized procedures for spaceflight experiment

Given that spaceflight experiments are costly endeavors, there is a need to robustly test protocols to be used by the crew and address potential experiment risks. With the completion of the WLA design and fabrication, we developed and tested a crew protocol to load animals into the NemaFlex-S device and achieve optimal animal occupancy in the pillared chambers. Instead of shipping the NemaFlex-S devices dry for spaceflight experiment, and priming them on ISS, which might lead to bubble formation as the liquid permeates the pillared chambers, we chose to prime the devices with deionized (DI) water on Earth and address any potential bubble issues prior to flight by conducting long-term device storage experiments. Below, we discuss our optimization efforts to develop a crew-friendly and time-efficient protocol for loading animals and our risk-mitigation strategy to have NemaFlex-S devices remain bubble-free.

**Fig. 5:**
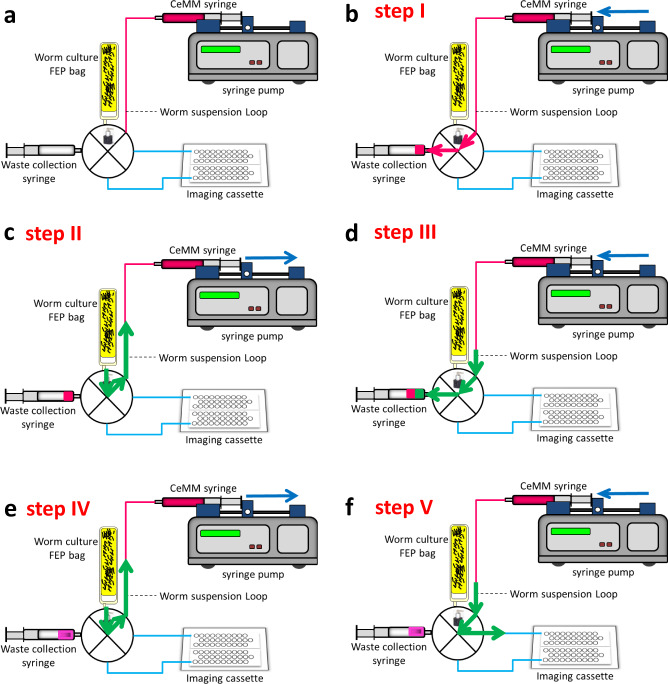
Schematic diagram of protocol of loading worms into NemaFlex-S device using the Worm Loading Apparatus. **a** Shows the schematic of the worm loading apparatus. (**b**) Step I: priming of the valve with CeMM, flow will be from CeMM media syringe to waste collection syringe. (**c**) Step II: aspirating a specified worm aliquot from worm culture bag to worm suspension loop. **d** Step III: priming of the valve with worm suspension, flow is from suspension loop to waste collection syringe. (**e**) Step IV: taking a specified worm aliquot (400 µL) from worm bag to suspension loop. (**f**) Step V: load the worms into A side of the NemaFlex-S device. Repeat step IV and step V to load the worms into the B side of the NemaFlex-S device.

The objective of the worm loading protocol involved replacing the DI water present in the pre-filled NemaFlex-S devices with CeMM, drawing a defined volume of worm suspension to inject into the chambers, and then pumping CeMM through the devices to remove progeny in the chambers. This objective was accomplished in multiple steps as shown schematically in Fig. 5 and Supplementary Table 2. Figure 5a shows the schematic of the WLA, with the worm suspension loop present underneath the distribution valve (see Fig. 4c). Since all the lines in the WLA board are primed with DI water during storage, therefore we need to prime the line and distribution valve with CeMM to remove DI water and any possible air pockets developed during the storage process. Therefore, in step I, 1 mL of CeMM was purged towards the waste collection syringe at 1000 mL/h as shown in Fig. 5b.

The next step involved priming with worm suspension because the distribution valve and ports have a dead volume that is filled with DI water during storage. Prior to performing this step, the culture bag was thoroughly mixed using a 3 mL syringe to ensure uniform distribution of animals. This process also removes any air trapped in the neck of the bag. Subsequently, the culture bag was manually spun to ensure bubbles collect at the far end of the bag. Immediately after this step, a clamp was used to prevent the air in the headspace reaching the valve port. Finally, the bag was connected to the worm loading port, and 400 µL of worm suspension was withdrawn from the worm culture bag (Fig. 5c) and subsequently transferred into the waste collection syringe at 400 mL/h (Fig. 5d).

Subsequent steps involve loading animals in the NemaFlex-S device with the two sides (NF-A and NF-B) being loaded sequentially. For this step as well, the culture bag was mixed and 400 µL of worm suspension was withdrawn at 400 mL/h (Fig. 5e). Then the valve was turned towards NF-A to replace the DI water in the microfluidic device with CeMM at 25 mL/h. Next, the 400 µL of worm suspension was aspirated to trap animals at the neck, followed by 1500 µL of CeMM at 25 mL/h to wash out the progeny (Fig. 5f). Overall, this procedure took ≈17 min to complete per imaging cassette (see Supplementary Table 2).

Next, we tested the efficacy of the crew-assisted loading protocol described above by keeping the animal density in the culture bags at 1000 adults/mL and maintaining the maximum push flow rate of animals into the chamber at 25 mL/h. We optimized the protocol by varying the flow rates for trapping the animals in the tapered necks since trapping is the rate-limiting step in the worm loading protocol. Additionally, we optimized the worm suspension volume aspirated from the culture bag, as the number of animals in the aspirated volume will determine the frequency of trapping and occupancy rate in the chambers. Our objective was to identify conditions that resulted in most chambers being occupied with 1 to 2 gravid adults to allow convenient software processing of pillar deflections.

**Fig. 6:**
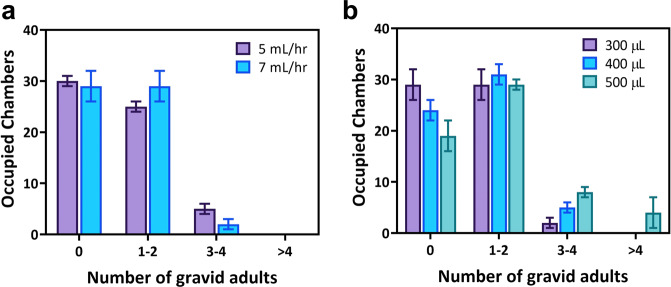
Optimization of parameters used for efficient loading of worms into NemaFlex-S device. **a** Effect of flow rate used for worm trapping in the tapered neck (Supplementary Table 3). **b** Effect of worm suspension volume (Supplementary Table 4). The density of animals in the culture bag was 1060 ± 160 adults/mL. Error bars represent standard deviation.

As shown in Fig. 6a, two different flow rates of 5 mL/h and 7 mL/h were used for trapping the worms in the tapered neck. The aspirated worm suspension volume was kept constant at 300 µL. We found that the number of chambers occupied by 1 to 2 gravid adults were relatively higher for 7 mL/h flow rate (29 ± 3 chambers) compared with 5 mL/h flow rate (25 ± 1 chambers). In addition, trapping at 7 mL/h reduces the total time of loading. Next, we optimized the worm suspension volume while keeping all other parameters constant. Three different volumes (300 µL, 400 µL, and 500 µL) of worm suspension were used to load the NemaFlex-S device. The total number of chambers occupied by gravid adults when using suspension volumes of 300 µL, 400 µL, and 500 µL were 31 ± 3, 36 ± 2, and 41 ± 3, respectively. Although the total number of chambers occupied with gravid adults was highest for 500 µL, it had only 29 ± 1 chambers occupied with 1-2 animals compared with 31 ± 3 chambers for 400 µL (Fig. 6b). Therefore, 400 µL of worm aliquot was used in the crew protocol for loading animals in the NemaFlex-S device.

### Long-term storage of NemaFlex-S device in WLA

Our need to conduct an 8-week long experiment with PDMS-based microfluidic devices raised two important concerns. First, since we primed the NemaFlex-S devices with DI water, there could be water loss due to PDMS being a porous substrate causing bubble formation. Second, the dissolved gases in the liquid might cause bubble formation in PDMS devices due to changes in local pressure or temperature or wettability, or geometrical conditions. Both these factors may cause bubble formation and make the NemaFlex-S devices unsuitable for clean images needed for optimal strength measurements. Therefore, there was a need to develop a mitigation strategy to address these potential risks.

In general, the PDMS layer is porous and hydrophobic in nature. The PDMS devices can be turned into hydrophilic by oxidized plasma bonding and it is reported that the hydrophilic nature of the device can be maintained for a longer duration of time if the device is submerged in water^48–50^. Placing the entire WLA board in water is practically impossible due to space/payload constraints. Therefore, we adopted an alternative approach to mitigate water loss and maintain the hydrophilic nature of devices by creating a humidified environment around the PDMS surfaces. We not only maintained the humid environment around the microfluidic chip, rather we keep the wet towel directly on top of the PDMS microfluidic device. Placing a wet towel directly on top of the PDMS layer is equivalent to submerging the device in water. A blue surgical towel was attached on top of the PDMS layer and soaked with 5 mL of DI water. Another piece of the towel was attached to the bottom side of the WLA platform and again soaked with 7.5 mL of DI water. Once these moist towels were attached, the entire WLA was kept inside a Bitran™ bag. The excess bag was folded over the WLA and then put into a secondary Bitran bag. Humidity sensors were placed inside the primary bag, and a 9-week storage experiment was conducted. We observed less than 1% loss in humidity during the test period indicating that our approach to preventing moisture loss and minimizing bubbles within NemaFlex-S devices was successful. This technique helps in maintaining the hydrophilicity of the microfluidic device and prevents the formation/nucleation of air bubbles in the NemaFlex-S devices.

### Ground testing of microfluidics-integrated hardware

Upon integration of the WLA with the NemaFlex-S device and optimization of the worm loading protocol, we piloted a ground study to measure the muscle strength of wild-type *C. elegans* using the multi-generational culture protocol. The objective of the ground testing was to demonstrate the capability to implement the multi-generational culture, loading protocol, imaging session, and analysis of the videos. This allowed us to assess risks associated with maintaining sterile culture over the 8 weeks of experiment, achieving efficacious worm loading from a mixed population, and observing consistency in strength measurement of gravid adults given that all gravid animals may not be of the same age.

**Fig. 7:**
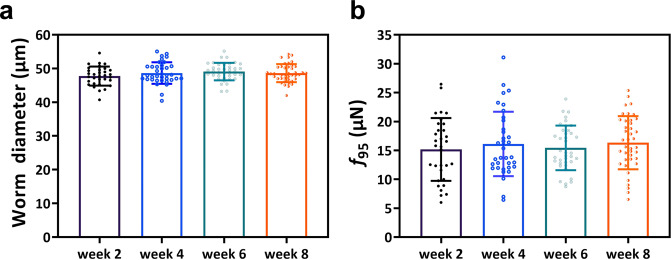
Measurement of muscle strength for wild-type *C. elegans* during multigenerational experiment. **a** Worm diameter over multiple generations. **b** Muscle strength over multiple generations. *n* = 32 for week 2, *n* = 36 for week 4, *n* = 38 for week 6, and *n* = 41 for week 8 (Supplementary Table 5). Error bars represent standard deviation. All the data pass the normality test. There is no significant difference between force values using one-way ANOVA with *P* = 0.8.

Figure 7 shows the results of this ground study. The worm diameter at mid-length was also measured to assess the homogeneity of gravid adults occupied in the chambers, which were visually selected for strength analysis based on the presence of eggs. As shown in Fig. 7a, the gravid adult body diameter did not show a statistical difference between animal groups that were assessed during each time point during the experiment, suggesting that the protocols for culture and loading providing a sufficient number of animals suitable for strength evaluation.

Next, we analyzed the 60-s videos acquired for each gravid adult. We identify the pillar with the maximal deflection in each image and generate a cumulative probability distribution with all the maximal deflections. We use the 95^th^ percentile of this maximal force distribution, referred to as *f*_*95*_, as a measure of muscle strength^30^. Since the worm body diameters at different time points were not significantly different from each other during this 8-week experiment, its effect on strength measurements was considered negligible. As expected, we did not find any statistical difference in the strength values for wild type on weeks 2, 4, 6, and 8 (Fig. 7b).

### Flight launch and on-orbit operation

In summary, this pilot study demonstrated that the experimental procedures developed for the microfluidics-integrated spaceflight hardware make the technology amenable to *C. elegans* muscle strength investigations on the ISS. Primary culture bags were prepared at Texas Tech University (TTU) with optimized worm densities (1000 Larve/mL) and shipped to Eastern Virginia Medical School (EVMS), Norfolk in a temperature control box. The culture bags were inspected for any possible contamination by the TTU team at EVMS upon arrival and incubated at 20^o^C until flight turnover. The NemaFlex-S chips were assembled on the WLA board at EVMS by the payload developer (BioServe Space Technology). Given the success of the pilot study, the microfluidics-integrated hardware and culture bags were flown to the ISS on Feb 20^th^, 2021, as part of the Northrop Grumman Cygnus NG-15 cargo mission. The same number of WLA boards and culture bags were prepared for ground control and shipped to TTU by mimicking the approximately same temperature profile used in the launch.

The culture bags were incubated at 20 °C in SABL, and the CeMM bags were stored at 4 °C after receiving them at the ISS. The worms were cultured for a week in microgravity conditions before conducting timepoint-1 activities. Astronauts Shannon Walker, Kathleen Rubins, and Michael Hopkins conducted the On-Orbit operation, with guidance provided to them by the authors of this study. The crew successfully inoculated the culture bags for subsequent time points by employing the culture protocol discussed in Sec. 2A. The worms were loaded into the NemaFlex-S chip, and the movement of worms in the NemaFlex-S chambers was recorded successfully.

The experiment went well for 2 generations (time points 1 and 2), and sufficient movies were recorded for muscle strength analysis. We saw a drastic decline in the animal density for time point 3 and 4 in worms cultured at ISS, whereas ground control cultures grew as expected. The reasons for the decline are unknown to us. Preliminary investigations suggest that it could be due to radiation, contamination, or maybe a combination of both, which is yet to be ascertained. We also froze worm cultures after NemaFlex experiments to conduct gene expression studies, enabling us in the future to correlate muscle strength changes to alterations in gene expression. No drying or glass cracks were observed in the NemaFlex-S device over long-time storage. Additionally, no leakage was observed while transferring the cultures from one bag to another and while loading them into the NemaFlex-S device. Thus, the microfluidics-integrated spaceflight hardware for muscle strength investigations in *C. elegans* has been successfully launched and operated onboard at the ISS. Hundreds of videos were collected along with frozen culture samples for gene expression analysis – this data is being currently analyzed and the results will be reported in the future.

## Discussion

With the human ambition of becoming multi-planetary species, there is a need to understand how the conditions of space (microgravity and radiation) affect the biology and physiology of species grown for multiple generations. Model organisms continue to play an important role in space biology investigations^51^. Conducting multi-generational studies in mammals on the ISS is a daunting task, however, the *C. elegans* model presents a unique opportunity for such investigations.

Compared to vertebrates, the *C. elegans* model has significant advantages including a fast generation cycle, small form factor, low payload mass, and translational relevance due to conserved molecular mechanisms. Despite these advantages, conducting successful experiments on the ISS is a major technological feat since it requires the design and integration of crew-interfacing user-friendly, and safe flight hardware and operational protocols. The focus of our study is not only the design of the NemaFlex-S device, but its integration into the Worm Loading Apparatus (WLA) that the crew can use to conduct experiments on the International Space Station (ISS). Below, we discuss the challenges of conducting multigenerational studies and the impact that our microfluidics-integrated space-flight hardware can have on space-biology investigations in *C. elegans*.

The major challenge for conducting multigenerational studies even in *C. elegans* is to identify suitable culture methods to breed them across generations while maintaining age-matched populations. The standard approach of culturing *C. elegans* for multiple generations on NGM plates that is typically done on Earth studies, is not suitable since (i) manual picking and transfer of animals to new plates to maintain synchronized populations is laborious and moreover, the open nature of the transfers represents a significant biohazard for ISS investigations. (ii) An additional constraint is the maintenance of defined bacterial population densities across the 8-week duration of our spaceflight experiment may present challenges since high bacterial density might limit oxygen availability for nematodes. To overcome these issues, we have cultured *C. elegans* inside FEP bags using chemical-defined liquid *C. elegans* maintenance media, which is being tested and used in the previous space mission’s^38,52^.

The power of the *C. elegans* model is the availability of a large number of muscle mutants that can potentially serve as biological controls. However, most of these mutants were obtained by standard culture on NGM plates and bacterial diet, and their viability in CeMM across multiple generations remains an open question. Identifying mutants grown in CeMM is challenging as they not only need to be viable, but also their growth rates need to match wild-type animals so that optimized density is available to load into the NemaFlex-S device. In the future, more studies need to be conducted to identify muscle mutants that grow well in CeMM across multiple generations. In our study, since we have wild-type animals growing on Earth, they serve as a biological control to compare against space-bred worms. Therefore, any changes in muscle strength could be compared with respect to ground control.

As summarized in Table 1, most flight studies conducted with *C. elegans* are confined to molecular analysis due to a lack of tools that can quantitate animal behavior, locomotion, and other physiological measures under the constraints imposed by spaceflight and crew time^13,14,17,19,25,53,54^. Development of new crew-compatible hardware is a major challenge since it requires close collaboration between scientists, payload developers, and space agencies; along with considerations of cost, upload mass, and foot-print requirements. Thus, most science-based investigations are limited by what hardware is available on the ISS.

Microfluidic devices present a unique opportunity to capture in vivo physiology data for small animals like *C. elegans* on the ISS^55,56^. However, there has been a lack of microfluidics integrated flight-ready hardware that is compatible with crew requirements and can report on muscle physiology. In the present study, we validated microfluidic integrated hardware on Earth and successfully launched it to the ISS. The library of videos acquired from the spaceflight study along with the gene expression data from multiple generations of animals will be a valuable resource to the scientific community that has been enabled by our integrated technology. The advancements offered by this hardware are as follows: (i) The microfluidic device is suitable for sorting the gravid adult animals of a specific size from a mixed culture and allowing the removal of progenies. (ii) The microfluidic device is integrated with a leak-proof cassette that can retain the waste generated during the loading of the worms, and therefore prevent contamination of the workstation. (iii) The worm loading apparatus helps in loading the animals in the microfluidic device with minimal effort from the crew. Astronauts need to simply click on the pump program and change the knob position on the distribution valve. (iv) Convenient recording of low or high-resolution videos of the animal inside the chambers allowing access to whole-organism phenotypic data.

As mentioned earlier, previous space missions were mostly limited to molecular analysis due to the lack of proper hardware. The integrated microfluidic hardware developed here is easy to use and opens new avenues for conducting behavioral and stress assays onboard ISS since the *C. elegans* model is widely accepted by the space biology community to understand the effects of short and long-term space missions. The hardware can be used for assessing the effect of environmental toxins, drugs, gut microbes, in-flight radiation, *etc*. The videos can be recorded based on the need of the study design and can be used to detect the changes in the behavior of the animal since behavioral alterations are an earlier indicator of toxic exposure than the death of the organism^57^.

The current hardware can also be used to study aging since the physiological and pathological changes occurring in space-flight conditions are reminiscent of accelerated aging. Indeed, how the spaceflight environment affects animal lifespan and healthspan is not well understood. Attempts have been made in the past to understand aging in *C. elegans* in space^14,58^. However, these studies do not preserve the identity of the worms and use biomarkers to inform on aging, rather than actual lifespan or healthspan measurements. The current hardware provides the basic solution to both problems as synchronized animals can be housed individually in the chambers and image-based phenotypic data can be obtained. Implementing some degree of automation to the current hardware could enable facile aging investigations on the ISS while reducing crew time.

The present study sets the stage for future space biology investigations on the ISS. Given that the *C. elegans* model is used in a variety of fundamental biology investigations ranging from behavior to neurobiology to aging, we anticipate that the NemaFlex-S devices used in this study will aid in such investigations. Additionally, our documented protocols for animal culture, animal loading, and the flow control system could be adapted to instrument a variety of *C. elegans*-based microfluidic devices already reported in the literature for spaceflight studies^43–46,59^. Successful completion of experiments with our technology over multigeneration in a microgravity environment opens the door for future discoveries using *C. elegans* as a model organism. Finally, the microfluidic technology developed in this work can also be of Earth benefit, since individuals can be monitored to understand how muscle atrophy occurs in a human disease setting and aging.

## Methods

### Worm culture preparation

Wild-type Bristol isolate (N2) worms were maintained in CeMM (Cell Guidance Systems, Cambridge, UK). To start culturing the animals in CeMM, agar-grown animals were bleached, and the eggs were introduced into the CeMM. The stock culture was then maintained by transferring aliquots of animals to fresh CeMM before cultures became starved. For the initiation of NemaFlex experiments, approximately 1000 larvae from a stock culture were transferred into an FEP bag (Saint Gobain Performance Plastics Corporation, Ohio, USA) containing 20 mL of CeMM. The FEP bags were stored in the incubator at 20 ± 1 °C. A mixed population of the well-fed gravid adult animals was used for all force measurement experiments. Every other week, 1 mL aliquots from the culture bag were transferred to a new bag containing 19 mL of fresh CeMM to make a total of 20 mL to initiate the new subcultures.

### Thrashing assay

To quickly assess the physical health of the animals, we performed a thrashing assay every other week. To measure the thrashing frequency, a 250-µL aliquot of worms was withdrawn from the culture bags and transferred into a Petri plate, and diluted with 2 mL of CeMM. The animals were left to acclimatize for 10 min. The number of body bends was manually counted under a dissecting microscope for 40 random individual worms for a period of 20 s at room temperature (20 ± 1 °C).

### Microfluidic device fabrication

The micropillar-based NemaFlex-S device was fabricated using a 2-step soft lithography process. The mold was fabricated in SU-8 2050 negative photoresist (Microchem) on a 4” silicon wafer as a substrate. First, a 20-µm tall photoresist layer was fabricated, which forms the boundary of the NemaFlex chamber. On top of this layer, a second layer of 80 µm height was fabricated with cylindrical holes that form the micropillars. This two-layer approach provides a total chamber depth of approximately 100 µm and creates deformable pillars of height 80 µm.

Polydimethylsiloxane (PDMS) devices of thickness 4.25 ± 0.25 mm thick were cast using Sylgard 184 part A (base) and part B (curing agent) (Dow Corning) 10:1 by weight over the SU-8 mold by curing for ∼2 h at 70 ± 1 °C. Inlet, outlet, and air vent holes were cored with a 1 mm hole puncher (Accuderm, Florida, USA). The devices were thoroughly cleaned with scotch tape to remove any dirt before bonding. The PDMS replica was then treated in an air-plasma cleaner (Harrick Plasma, Ithaca, NY) for the 90 s and bonded to a 2”×3” glass slide. Bonding was done while ensuring the pillars did not collapse or deform. The bonded devices were immediately kept in the oven for 10 min at 70 ± 1 °C. Devices were then treated with 5 wt% Pluronic F127 (Sigma-Aldrich) for 30 min to prevent any bacterial build-up and also to help reduce bubble formation during worm loading. After incubation, excess Pluronic was removed by washing the devices with DI water. The Pluronic-treated devices were soaked in DI water overnight at 20 ± 1 °C to release any air bubbles from the device. The PDMS devices were then integrated into WLA and kept in double Bitran bags and stored at ambient temperature until the day of the experiment.

### Image acquisition and processing

The worms were loaded into NemaFlex-S chambers using the procedure described in the main text. The animals were allowed to habituate in the micropillar arena for approximately 10 min before imaging. One-minute-long videos of crawling worms were acquired with Nikon inverted microscope (Eclipse TS 100) at 4X magnification with a field of view of 1920 × 1080 pixels. All videos were recorded at a temperature of 22 ± 1 °C. The recorded movies were processed offline using custom routines written in MATLAB (Mathworks, R2018b) for quantitation of pillar displacements as discussed by Rahman et al.^30^. Worm diameters were measured manually using ImageJ 1.48 v.

### Reporting summary

Further information on research design is available in the Nature Research Reporting Summary linked to this article.

## Supplementary information

Supplementary Information

Supplementary Video

Reporting Summary

## Data Availability

All data generated or analyzed during this study are included in the main text and its supplementary information file. The video files and images used in this study are available from the corresponding author upon reasonable request.

## Code availability

The custom routines are written in MATLAB (Mathworks, R2018b) for quantitation of pillar displacements and can be accessed at https://github.com/VanapalliLabs/NemaFlex. The repository provides the code and instructions for processing NemaFlex movies, where C. elegans deflect micropillars.
