# Supplementary material for: Microfluidics-integrated spaceflight hardware for measuring muscle strength of Caenorhabditis elegans on the International Space Station
Source: NPJ Microgravity. 2022 Nov 7;8:50. doi: 10.1038/s41526-022-00241-4 (PMC9640571; doi:10.1038/s41526-022-00241-4)
Supplement: Supplementary file 1 — Supplementary Information [file 41526_2022_241_MOESM1_ESM.pdf]

Microfluidics-integrated spaceflight hardware for measuring muscle strength of *Caenorhabditis elegans* on the International Space Station

Purushottam Soni,[1*] Taslim Anupom,[2*] Leila Lesanpezeshki,[1] Mizanur Rahman,[1] Jennifer E. Hewitt,[1] Matthew Vellone,[3] Louis Stodieck,[3] Jerzy Blawzdziewicz,[4] Nathaniel J. Szewczyk,[5] and Siva A. Vanapalli,[1‡]

[1]Department of Chemical Engineering, Texas Tech University, Lubbock, TX 79409 USA. [2]Department of Electrical Engineering, Texas Tech University, Lubbock, TX 79409 USA. [3]BioServe Space Technologies, Boulder, CO 80303 USA. [4]Department of Mechanical Engineering, Texas Tech University, Lubbock, TX 79409 USA. [5]Ohio Musculoskeletal and Neurological Institute and Department of Biomedical Sciences, Ohio University, Athens, OH 43147, USA

*Equal author contribution

‡ Corresponding author E-mail: siva.vanapalli@ttu.edu

Supplementary Table 1: Swim-induced thrashing frequency of gravid adults as a measure of their locomotory health. The number of body bends was manually counted under a dissecting microscope for 40 random individual worms for a period of 20s at room temperature. There is no significant difference between thrashing frequency as calculated by one-way ANOVA, $P \geq 0.7$.

Time Point	thrashing frequency (1/min)
Week 2	108.3±22.6
Week 4	109.9.3±16.4
Week 6	110.2±19.6
Week 8	109.3±15.7

Supplementary Table 2: Breakup of time required for loading the worms into the NemaFlex-S Chamber. It approximately takes 17 minutes to load one NemaFlex-S device.

step	Operational steps	Time required	Manual/Automatic
1	Turn distribution valve towards waste syringe	2 sec	manual
2	infuse 1mL CeMM to waste syringe @ 1000 mL/hr	4 sec	automated
3	Mix worm culture bag, and connect FEP culture bag to distribution valve port	30 sec	manual
4	Turn valve to FEP culture bag	2 sec	manual
5	Withdraw 400 µL worm aliquot @ 400 mL/hr from culture bag	4 sec	automated
6	Turn valve toward waste collection	2 sec	manual
7	Infuse the worm aliquot to waste @ 400 mL/hr	4 sec	automated
8	Disconnect FEP culture bag and mix it well and connect back to distribution valve	45 sec	manual
9	Turn valve to FEP culture bag and hit loading protocol on pump	2 sec	manual
10	Withdraw 400 µL worm aliquot @ 400 mL/hr from culture bag	4 sec	Automated
11	Turn valve towards NemaFlex-S A side	2 sec	manual
12	Load worms into chambers and wash it with 1500µL CeMM @ 25 ml/hr	7 min 10 sec	Automated
13	Repeat step 8 to 12 for NF-B	8 min	

Supplementary Table 3: Effect of flow rate used for worm trapping on loading efficacy at a fixed worm suspension volume of 300 μL. The density of animals in the culture bag was 1060±160 adults/mL.

Number of worms in a chamber	Number of chambers occupied at 5mL/hr	Number of chambers occupied at 7 mL/hr
0	30±1	29±3
1 - 2	25±1	29±3
3 - 4	5±1	2±1
More than 4	0	0

Supplementary Table 4: Effect of worm suspension volume on the loading efficacy at a fixed trapping flow rate of 7mL/hr. The density of animals in the culture bag was 1060±160 adults/mL.

Number of worms in a chamber	Number of chambers occupied for 300 μL aliquot	Number of chambers occupied for 400 μL aliquot	Number of chambers occupied for 500 μL aliquot
0	29±3	24±2	19±3
1 - 2	29±3	31±2	29±1
3 - 4	2±1	5±1	8±1
More than 4	0	0	4±3

Supplementary Table 5: Summary of the worm diameter and force value for different time points during a multigenerational study on the ground.

Time Point	Sample Size	Worm diameter (μm)	f_{95} (μN)
Week 2	32	47.76±2.78	15.17±5.34
Week 4	36	48.64±3.16	16.12±5.49
Week 6	38	49.08±2.57	15.51±3.70
Week 8	41	48.63±2.65	16.33±4.54

Supplementary Movie 1: Worm trapping and loading into the NemaFlex-S chamber.
